# Efficacy and safety of repetitive Transcranial Magnetic Stimulation and transcranial Direct Current Stimulation in memory deficits in patients with Alzheimer's disease: Meta-analysis and systematic review

**DOI:** 10.1016/j.ijchp.2024.100452

**Published:** 2024-03-01

**Authors:** Sara M. Fernandes, Augusto J. Mendes, Pedro F.S. Rodrigues, Ana Conde, Magda Rocha, Jorge Leite

**Affiliations:** a CINTESIS@RISE, CINTESIS.UPT, Portucalense University, 4200-072 Porto, Portugal; b Laboratory of Neuroimaging of Aging (LANVIE), University of Geneva, Geneva, Switzerland; c Geneva Memory Center, Department of Rehabilitation and Geriatrics, Geneva University Hospitals, Geneva, Switzerland; d Brain@Loop Lab

**Keywords:** Alzheimer, AD, rTMS, tDCS, Memory

## Abstract

Repetitive transcranial magnetic stimulation (rTMS) and transcranial direct current stimulation (tDCS) are two of the most used non-pharmacological interventions for Alzheimer's Disease (AD). However, most of the clinical trials have focused on evaluating the effects on global cognition and not on specific cognitive functions. Therefore, considering that memory loss is one of the hallmark symptoms of AD, we aim to assess the efficacy and safety of tDCS and rTMS in memory deficits. For that, multilevel random effect models were performed considering the standardized mean difference (SMD) between active and sham stimulation. A total of 19 studies with 411 participants demonstrated positive effects in memory after tDCS (SMD=0.20, *p* = 0.04) and rTMS (SMD=0.44, *p* = 0.001). Subgroup analysis revealed that tDCS had greater efficacy when administered in temporal regions (SMD=0.32, *p* = 0.04), whereas rTMS was superior when applied in frontal regions (SMD=0.61, *p* < 0.001). Therefore, depending on the brain region of stimulation, both interventions produced a positive effect on memory symptoms in AD patients. Finally, the safety of both techniques was observed in the AD population after the reporting of almost no serious events.

## Introduction

Alzheimer's Disease (AD) is the most common cause of dementia worldwide and is characterized by a gradual and slow decline in memory and other cognitive processes capable of interfering with daily life (Sanches et al., 2021). AD pathology is defined by extracellular accumulation of amyloid-β (Aβ), strings of hyperphosphorylated Tau proteins accumulating inside neurons, and consequent neurodegeneration (Jack et al., 2016). Pathological changes in the brain begin long before the first signs of memory loss. For this reason, when diagnosed (usually when the first symptoms appear), the neuropathological process is already advanced (Trejo-Lopez et al., 2021).

Interventions targeting AD primarily aim to prevent cognitive decline through pharmacological interventions and cognitive training. However, there is a growing need to investigate the effectiveness and safety of non-pharmacological therapies as potential preventive measures against the onset or progression of AD symptoms. At this level, non-invasive brain stimulation (NIBS) is proposed as a promising non-pharmacological therapeutic option for AD, where repetitive transcranial magnetic stimulation (rTMS) and transcranial direct current stimulation (tDCS), are among the most extensively studied therapies for this disease. Some studies testing the effect of rTMS and tDCS showed a positive effect of these methods on the enhancement of global cognition in patients with AD (Sanches et al., 2021). tDCS is based on the application of a weak constant electrical current (1–2 mA) through an *active (*anode) and a *return* electrode (cathode) (Nitsche & Paulus, 2000). The cortical excitability of the stimulated brain region can be modulated by the electrical current, which changes the likelihood of generating an action potential (Rahman et al., 2013). As a result, anodal stimulation is suggested to increase cortical excitability, whereas cathodal stimulation decreases cortical excitability (Stagg & Nitsche, 2011). This is of particular interest for cognitive enhancement (Coffman et al., 2014) as well as for clinical application in neurological and psychiatric disorders (Fregni et al., 2021).

On the other hand, rTMS is a non-invasive brain stimulation technique using a brief-lasting magnetic field to painlessly convey electrical current into a brain cortical area (Siebner et al., 2022). Such current has sufficient intensity to trigger action potentials in the stimulated region, allowing the propagation of the signal towards connected regions. Consequently, short rTMS patterns have been used to change or synchronize frequency-specific rhythmic oscillations of neurons to modulate specific cognitive operations (Thut et al., 2011). The application of rTMS has demonstrated the ability to induce two distinct effects based on the frequency of the pulses. Specifically, when rTMS is administered at frequencies of approximately 5 Hz and higher, it has been observed to provide a sustained excitatory effect. Conversely, when rTMS is employed at frequencies of 1 Hz and lower, it has been found to elicit an inhibitory effect (Fitzgerald et al., 2006). Therefore, in line with tDCS, rTMS suggests its usefulness to enhance cognitive abilities (Luber & Lisanby, 2014), as well as a therapeutical tool for several clinical conditions including AD (Lefaucheur et al., 2020).

High-frequency rTMS and anodal tDCS delivered for at least 2 weeks have shown improvements in cognitive function in patients with AD, maximizing performance and containing the progression of cognitive decline (Sanches et al., 2021). Nonetheless, studies approaching both techniques used different methods and stimulation parameters, which can complicate the understanding of the non-invasive brain stimulation techniques in AD (Holczer et al., 2020). Moreover, studies with rTMS and tDCS have demonstrated positive results in improving the general cognitive functioning of patients with AD, but their specific effect on several cognitive functions is not clear. This is the case with memory impairment, which is one of the earliest and most common symptoms of AD (Scheltens et al., 2016), even though research studies often do not prioritize the evaluation of this specific function. Studies examining the impact of tDCS/rTMS on AD primarily assess global cognition using tools such as Mini-mental state examination (MMSE), Montreal Cognitive Assessment (MOCA), Clinical Dementia Rating (CDR), or Alzheimer's Disease Assessment Scale–Cognitive Subscale (ADAS-Cog), which hinder a proper evaluation of specific cognitive functions such as memory.

For that, we aimed to evaluate the literature about the efficacy of rTMS and tDCS on the enhancement of memory deficits in patients with an AD or probable AD diagnostic. Furthermore, although both techniques have been demonstrated to be safe procedures in different populations (Bikson et al., 2016; Rossi et al., 2021), a considerable number of studies do not evaluate the relevant adverse effects (AEs). Therefore, we also intend to evaluate the safety of both techniques in AD patients by analyzing the AEs.

## Methods

The systematic review and meta-analysis's various procedures were conducted accordingly to the recommendations in the Preferred Reporting Items for Systematic Reviews and Meta-Analyses (PRISMA; Moher et al., 2009). Our study was registered on the international prospective register of systematic reviews (PROSPERO) with reference number CRD42022349579.

### Literature search and study selection

The literature search was performed in PUBMED, Web of Science, and Scopus using a combination of tDCS, rTMS, memory, and Alzheimer's disease (all the terms and search strategies are reported in Table A in Supplementary Materials). The screening phase was performed in the Abstrackr (Wallace et al., 2012), in which each study was screened by two researchers independently. In case of disagreements, a third researcher resolved the conflict. The eligibility evaluation comprised the following inclusion criteria: i) randomized controlled trials (RCTs), ii) rTMS or tDCS groups as the intervention groups, iii) studies with a sham-controlled condition, iv) memory evaluation as outcome, v) sample of the study with an AD or probable AD diagnostic, and vi) studies written in English. Case-studies, systematic reviews, meta-analyses, or studies with other cognitive function evaluation were excluded. When there were several publications with the same cohort, we selected the study with a larger sample size. Additionally, global cognition scales, such as the Mini-Mental State Examination (MMSE) and Montreal Cognitive Assessment (MoCA), were not considered because they represent a composite score of different cognitive functions, only specific evaluations in memory were included. All studies' eligibility was initially evaluated using the abstract, and then, in a subsequent phase, using the full-text manuscript.

### Data extraction

The following information was extracted from each study: first author, year of publication, study design (e.g., crossover, parallel), number of subjects analyzed (i.e., excluding dropouts or outliers), cognitive training with stimulation (i.e., yes or no), safety evaluation (e.g., frequency of adverse effects), medication, diagnostic criteria, brain region of stimulation (i.e., anode, cathode, or coil location), tDCS parameters (i.e., intensity, density, and duration), rTMS parameters (i.e., intensity, frequency, and number of pulses), number of sessions (e.g., single or multi-session), and method of memory assessment (e.g., Rey Auditory Verbal Learning Test, n-back). The statistical results to extract were the mean and standard deviation of memory evaluation scores in active and sham groups for baseline, post-intervention, and follow-up. The Web Plot Digitizer (Rohatgi, 2017) was used to extract the statistics from the graphs if the data was not presented in text or tables. At last, an email was sent to the corresponding author requiring the required information if the necessary statistical data was missing in any format.

### Statistical analysis

The statistical analysis was performed in the memory outcomes using R (R Core Team, 2018) with the *metafor* package (Viechtbauer, 2010; metafor Version 4.0–0, released on 2023–03–19).

#### Multilevel meta-analysis, subgroup, and moderator analysis

The random-effect multilevel models were performed due to the high number of comparisons within some studies. As a result, the model considered the within variance for each study with multiple comparisons given that the individual effect sizes were nested within the corresponding study. For that, the extracted statistical data was used to compute the average change between baseline and post-intervention/follow-up as well as the pooled standard deviation between both measures for each study. Using these statistical metrics, the effect size between active and sham stimulation was determined using the unbiased Hedges' g (Hedges, 1981).

The multilevel models were performed independently for tDCS and rTMS since they share distinct neurophysiological mechanisms of action (Cochrane, 2019). To assess the short- and long-term effects of the stimulation on memory capacity, independent meta-analyses were conducted between the pos-intervention and follow-up. The follow-up analysis was only carried out in rTMS given that the tDCS studies lacked sufficient data (i.e., only one study reported follow-up evaluation). The Cochrane's Q test was performed to evaluate heterogeneity between studies. See Table B in Supplementary Materials for the labels in the forest plots.

Moderator and subgroup analysis were performed for the continuous and categorical moderators respectively, namely: brain region stimulation, intensity (for tDCS and rTMS), density, rTMS frequency, number of pulses, duration, study design, cognitive training, number of sessions, type of memory, and type of stimuli in the memory evaluation. The type of memory was categorized into short-term memory (STM), long-term memory (LTM), association memory (AM), working memory (WM), and recognition memory (RM) based on the task used to evaluate the memory. Additionally, the type of stimuli utilized in the memory assessment was also tested (e.g., letters, words, figures).

#### Influential analysis and publication bias

The leave-one-out method was employed to conduct the influential analysis. Given that we conducted a multilevel meta-analysis that allowed the inclusion of several comparisons from each study, this method allowed the meta-analysis to be recalculated by removing all the comparisons from a study each time (Viechtbauer & Cheung, 2010).

Egger's regression test and funnel plots were used to evaluate the publication bias (Egger et al., 1997). However, given that we are considering several measures from the same study, this heterogeneity within the study might influence the publication bias result. For that, all comparisons were displayed using funnel plots with estimates against their standard errors, with the colors denoting the study. On the other hand, Egger's regression test was a multilevel meta-regression with the standard errors (SE) of the effect size estimates as predictors. Thus, even when the variation between and within studies is accounted for, a significant effect on the SE suggests an asymmetry in the funnel plot (Fernández-Castilla et al., 2021).

### Safety evaluation

The safety of tDCS and rTMS in the AD population was evaluated based on the AEs reported in each study between active and sham stimulation.

### Risk of bias

The risk of bias was assessed by seven criteria, namely, random sequence generation, allocation concealment, selective reporting, other sources of bias, participants blinding, raters blinding, and lack of outcome data (Higgins et al., 2011). Each criterion was classified as “high risk”, “low risk” or “unclear” by two independent raters. A third researcher resolved the conflict in case of any mismatch between raters. The traffic light plots were done with the *robvis* package in R (McGuinness & Higgins, 2021); Version 0.3.0, released on 22–11–2019).

## Results

**Fig. 1:**
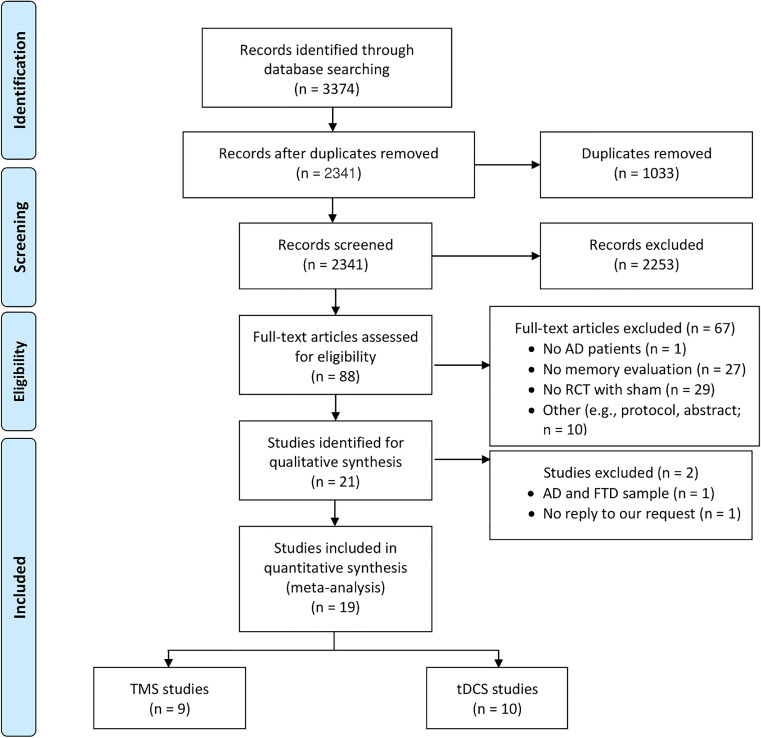
PRISMA flow diagram illustrating the literature search and inclusion process.

The abstract screening comprised 2341 studies, resulting in the exclusion of 2253 studies and the inclusion of 88 studies. At this point, a third reviewer resolved 6.7 % of the disagreements between the reviews from the two reviewers. After that, 21 papers were chosen for full-text screening; however, two of them contained a sample of AD and frontotemporal dementia, and one of them lacked statistical data. The primary reasons for eliminating such records were the absence of a memory assessment or their lack of being randomized controlled trials with a sham condition. Out of 3374 original records, a total of 19 studies were included; nine of these studies assessed the effects of rTMS, while 10 studies used tDCS (Fig. 1). See Table C in Supplementary Materials for the list of studies included in the current meta-analysis.

### rTMS studies

#### Study characteristics

The nine studies with rTMS comprised a total of 300 participants with AD. Seven (out of nine) studies had a parallel design with multiple sessions of rTMS. The stimulation was mostly applied in frontal areas (five studies), whereas three studies evaluated rTMS in parietal regions and only one in the cerebellum. Most studies applied rTMS alone given that only two studies combined rTMS with cognitive training (see Table D in Supplementary Materials). All the studies performed a baseline memory assessment. Lastly, six studies evaluated memory in follow-ups, and one study was only included in the follow-up meta-analysis (Wei et al., 2022) given that the cohort used in the post-intervention evaluation was the same as another study (Jia et al., 2021) but with a lower sample size.

#### Multilevel meta-analysis, subgroup, and moderator analysis

**Fig. 2:**
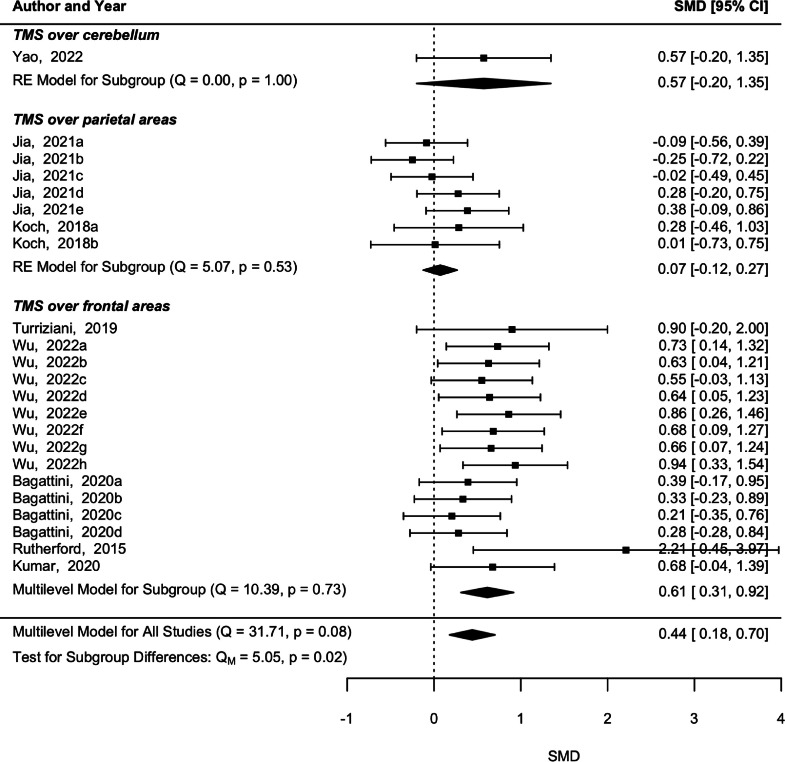
Forest plot with pooled effect estimate from post-rTMS evaluations and underlying subgroup analysis concerning the brain region of stimulation.

The pooled effect estimate from the eight studies did not show a significant heterogeneity (p = 0.08, *Q* = 31.71), and a significant effect was found on memory skills immediately after rTMS (*p* = 0.001, SMD = 0.44, 95 % CI [0.18 0.7]) (Fig. 2). Subgroup analysis by the brain region of stimulation revealed a significant effect in memory after rTMS over frontal regions (*p* < 0.001, SMD = 0.614, 95 % CI [0.31 0.92]) without a significant heterogeneity among comparisons (*p* = 0.733, *Q* = 10.39). The studies applying rTMS over parietal areas did not present significant heterogeneity (*p* = 0.535, *Q* = 5.071), nor a significant effect in memory (*p* = 0.472, SMD = 0.074, 95 % CI [−0.12 0.27]). The moderator analysis revealed that rTMS intensity is a significant moderator in memory abilities (*p* < 0.001, SMD = −0.02, 95 % CI [−0.03 −0.01]) suggesting a higher effect in lower-intensity stimulation. However, this effect is highly influenced by Wu et al. (2022) who applied a 70 % rTMS and Jia et al. (2021) who applied a 105 % rTMS. All the other moderators were not statistically significant (*p* > 0.05).

**Fig. 3:**
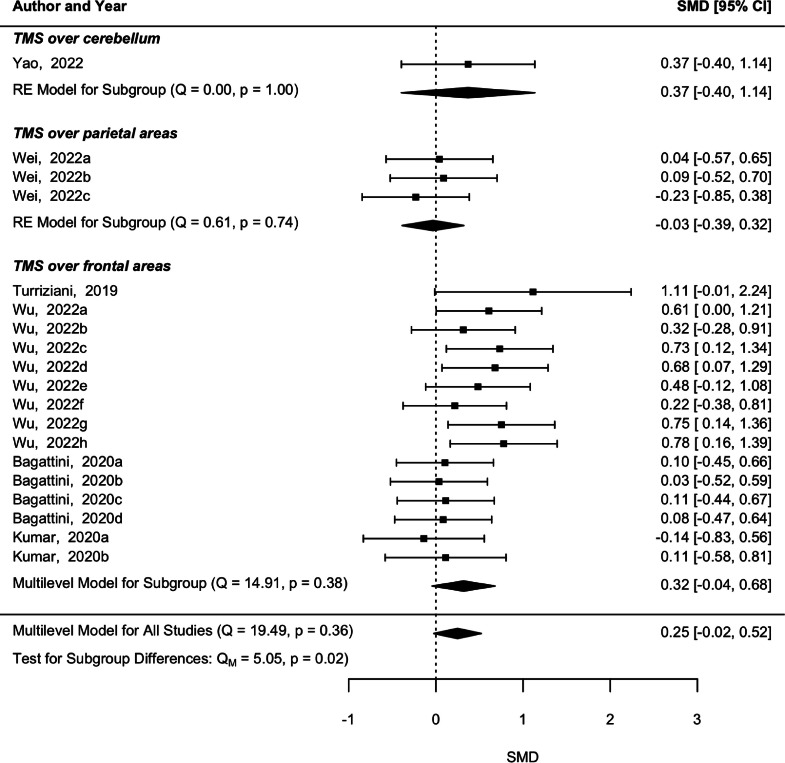
Forest plot with pooled effect estimate from the follow-up evaluations after rTMS and underlying subgroup analysis concerning the brain region of stimulation.

Regarding the follow-up evaluations, the heterogeneity between the five studies was not statistically significant (*p* = 0.36, *Q* = 19.49), however only a trend for memory abilities improvement was shown (*p* = 0.07, SMD = 0.25, 95 % CI [−0.02 0.52]) (Fig. 3). The subgroup analysis revealed that studies applying rTMS over frontal regions with follow-ups did not reveal a significant heterogeneity (*p* = 0.39, *Q* = 14.91) and the marginal significant effect was maintained in memory skills follow-ups after rTMS over frontal regions (*p* = 0.09, SMD = 0.32, 95 % CI [−0.04 0.68]). The subgroup analysis of follow-ups after rTMS over parietal and cerebellar areas was not performed because there was only one study in each. Moderator analysis also revealed a significant effect of rTMS intensity (*p* = 0.001, SMD = −0.016, 95 % CI [−0.03 −0.01]). This is the same effect observed in the post-intervention analysis, namely a higher effect in lower-intensity stimulation. The other moderators revealed statistically non-significant results (*p* > 0.05).

#### Influential analysis and publication bias

The leave-one-out method revealed that the pooled effect estimate from post-rTMS evaluation was not influenced particularly by any of the studies. However, in the follow-up analysis, the pooled effect was influenced by one study (Yao et al., 2022), in which rTMS was applied over the cerebellum. When this study was removed from the multilevel model, the trend turned into a statistically significant result (*p* = 0.04, SMD = 0.321, 95 % CI [0.01 0.63]), suggesting a long-term effect in terms of memory, for rTMS applied over frontal and parietal areas.

The publication bias analysis performed in the post-intervention rTMS showed an asymmetry in the funnel plot based on the significant result of SE in the Egger's regression test (*p* = 0.02, *z* = 2.32). However, the data outside the 95 % CI is mostly observed on the left side of the plot, which suggests lack of publication bias. Considering the data for follow-ups, the funnel plot follows a symmetry as shown in the multilevel regression Egger's test (*p* = 0.17, *z* = 1.38). The funnel plots are represented in Figure A of Supplementary Materials.

### tDCS studies

#### Study characteristics

The tDCS effects on memory were studied in 10 studies with a total of 214 participants with AD. The designs used were five parallel and five crossover studies. Five studies (out of 10) applied tDCS over frontal areas, three over temporal areas and two tested both stimulations. Moreover, two of these studies performed an additional cathodal stimulation, which was not considered in the current analysis due to the potential antagonistic effect in comparison with anodal stimulation (Cochrane, 2019). The tDCS was mainly applied alone because only two studies tested tDCS coupled with cognitive training (see Table E in Supplementary Materials). Regarding memory assessment, only one study did not provide a baseline evaluation (Cochrane, 2019). Furthermore, follow-up assessments were only performed in one study, which precluded the assessment of the long-term effects.

#### Multilevel meta-analysis, subgroup, and moderator analysis

**Fig. 4:**
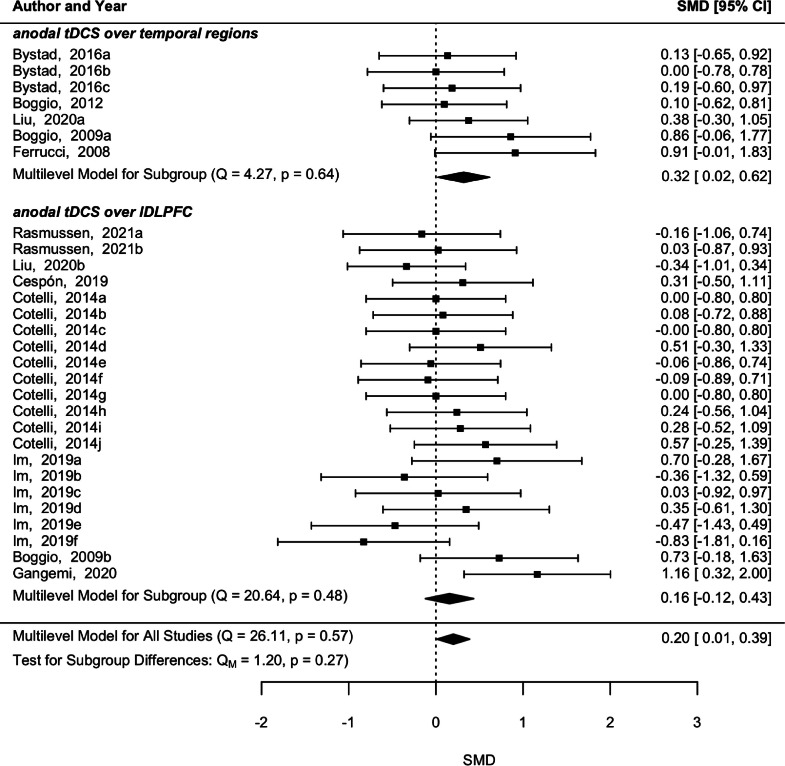
Forest plot with pooled effect estimate from post-tDCS evaluations and underlying subgroup analysis concerning the brain region of stimulation.

The 10 studies studying tDCS did not reveal significant heterogeneity among them (p = 0.567, *Q* = 26.11) and a pooled significant effect after tDCS was found for memory (*p* = 0.04, SMD = 0.20, 95 % CI [0.01 0.39]) (Fig. 4). Anodal tDCS over temporal regions also showed a significant improvement in memory abilities (*p* = 0.04, SMD = 0.32, 95 % CI [0.02 0.62]) without a significant heterogeneity among comparisons (*p* = 0.64, *Q* = 4.27). On the other hand, anodal tDCS over left dorsolateral prefrontal cortex (DLPFC) did not reveal a significant effect in memory (*p* = 0.27, SMD = 0.16, 95 % CI [−0.12 0.43]), nor significant heterogeneity (*p* = 0.48, *Q* = 20.64).

The subgroup analysis in types of memory revealed that the tDCS effect was only significant when recognition memory was assessed (*p* = 0.04, SMD = 0.49, 95 % CI [0.02 0.97]). Moreover, a significant effect of the number of tDCS sessions was also found (*p* = 0.049, SMD = −0.09, 95 % CI [−0.17 0]), thus suggesting that studies with one session had a larger effect size. All other moderators were not statistically significant

#### Influential analysis and publication bias

The influential analysis suggested the instability of the pooled effect estimate from post-tDCS evaluation because the significant effect became marginally significant each time one of four studies was removed (Boggio et al., 2009; Cespón et al., 2019; Cotelli et al., 2014; Gangemi & Fabio, 2020). Finally, the funnel plot suggests a lack of publication bias due to its symmetry (Figure A of Supplementary Materials) and its non-significant result in Egger's regression test (*p* = 0.58, *z* = 0.56).

### Risk of bias

The risk of bias was mostly labeled as “unclear” or “low risk” following the criteria of the Cochrane Risk of Bias tool (Higgins et al., 2011). The domains mostly evaluated with “Unclear” were randomization (4 out of 9 in rTMS; 7 out of 10 in tDCS), allocation concealment (7 out of 9 in rTMS; 7 out of 10 in tDCS), and participant blinding (9 out of 9 in rTMS; 10 out of 10 in tDCS). Most studies did not report the exact randomization process and how they concealed their allocation sequence. Moreover, the participant blinding was not assessed using the proper questionnaires (Bang et al., 2010). On the other hand, selective reporting was labeled as “low risk” in all studies except one tDCS study, in which data from a single memory task was retrieved, although another task was also performed. There were only two studies with a “high risk” of bias, one in tDCS and another in rTMS, whereas all the others were labeled as “low risk”. Both “high risk” studies showed a baseline imbalance in sociodemographic characteristics between the active and sham group. The rater blinding was evaluated mostly as “low risk” (8 out of 9 in rTMS; 8 out of 10 in tDCS). Finally, only one tDCS study was labeled as “high risk” considering the attrition bias, whilst all the other studies were labeled as “low risk”. Overall, the tDCS studies showed higher risk of bias in comparison with the ones with rTMS. The traffic light plots with the risk of bias are represented in Figures B, C, D, and E in Supplementary Materials.

### Safety assessment

A total of 12 studies evaluated safety, specifically six studies during or after the use of tDCS and six for rTMS. Most AEs reported were minor and related to scalp sensations, namely tingling or discomfort. In the context of tDCS research, six out of ten studies evaluated the safety of the technique. Most of the studies, specifically five out of six, did not report any adverse effect (Boggio et al., 2009, 2012; Bystad et al., 2016; Gangemi & Fabio, 2020; Rasmussen et al., 2021). However, one study reported itchiness (52.9 %), tingling (31.4 %), discomfort (13.7 %), and a burning sensation (13.7 %), even though tDCS was tolerable for all participants (Liu et al., 2020).

On the other hand, it is noteworthy that a majority of rTMS studies, specifically six out of nine, assessed the AEs. Participants from two studies did not report any major adverse effects during or after rTMS (Bagattini et al., 2020; Yao et al., 2022). In one of the studies reporting AEs, eight out of 47 participants reported painful scalp sensations, or eyelid twitches, and tinnitus (starting from the most prevalent to the least prevalent) in active and sham stimulation; however, all participants stated that the events were tolerable (Wu et al., 2022). Likewise, another study with repetitive paired associative stimulation (rPAS) reported 11 out of 16 AEs after active stimulation (two of them related with sleep), whereas seven out of 16 also reported after sham rPAS (Kumar et al., 2020). All the previous AEs were minor and included headache, pain/discomfort, fatigue and frustration in both groups. However, in one study, rTMS was not tolerated by two participants in the active group (5 %) and one in sham group (3 %) (Jia et al., 2021). One of the participants reported persistent scalp discomfort, while the other two reported transient fatigue. All the other participants did not report serious adverse effects (Jia et al., 2021).

## Discussion

Our study showed that rTMS over frontal regions and tDCS over temporal regions can improve memory abilities in people with AD. However, the prolonged effect of rTMS showed a statistical tendency towards enhancing memory, whereas the long-term effects of tDCS could not be assessed due to insufficient data. Finally, it was noted that both techniques demonstrate safety in the population affected by AD, with predominantly minimal side effects.

### rTMS

The primary factor contributing to the enhancement of memory in individuals with AD using rTMS was found to be the frontal stimulation interventions. These interventions were conducted in five out of the nine studies that examined the effects of rTMS. The improvement of memory of subjects with AD by frontal rTMS is in line with previous meta-analyses showing improved global cognition after rTMS (Teselink et al., 2021; Xiu et al., 2023; Zhang et al., 2022). The region of stimulation with better results in global cognition was the DLPFC, which is a core region in working memory (Barbey et al., 2013) and consequently involved in long-term memory formation (Blumenfeld & Ranganath, 2006). Not surprisingly, all the studies included in our subgroup analysis of frontal rTMS targeted the DLPFC. A prior investigation has already demonstrated that rTMS targeting the DLPFC can enhance memory abilities in individuals diagnosed with mild cognitive impairment and AD (Chou et al., 2022).

Interestingly, subjects with AD exhibit less neuroplasticity in the DLPFC region as compared to controls, which is associated with impaired working memory (Kumar et al., 2017). The diminished cortical plasticity might be due to the synaptic damage within networks involving cortical and subcortical regions, which are degenerated during AD progression (Walsh et al., 2017). A recent study demonstrated that following a six-week program of 10 Hz rTMS over the left DLPFC, patients with AD showed a modest improvement in global cognition alongside an increase in neuroplasticity (Li et al., 2021). Likewise, one session of high-frequency rTMS over left DLPFC was also capable of modulating cerebral blood flow in the stimulation region and underlying areas involved in the default mode network (DMN) (Shang et al., 2018). Hence, our results combined with previous literature on brain functioning highlight the significance of DLPFC as a target area for memory enhancement in subjects with AD using rTMS.

On the other hand, the follow-up memory evaluations were conducted in just six out of the nine trials that were included in our analysis. The follow-up memory scores in the six studies revealed marginally significant improvement (*p* < 0.1). Nevertheless, the influential analysis revealed that the effect, which was marginally significant, attained statistical significance (*p* < 0.05) after excluding Yao et al. (2022) using the leave-one-out technique. Our findings are in line with a previous meta-analysis that showed the potential for multi-session rTMS to elicit effects lasting up to 12 weeks in cognitive functions (Chou et al., 2022), despite demonstrating a smaller effect size when compared to the post-rTMS evaluation. Another trial testing 10 Hz rTMS for five weeks in AD patients observed an enhancement in ADAS-Cog until six months after the stimulation, even though this improvement was only detected in those who had the most favorable response to the treatment (Nguyen et al., 2017). Likewise, a prolonged effect of rTMS is also observed in neurophysiological markers such as the modulation of cortical excitability after the stimulation (Pascual-Leone et al., 1994). In a recent randomized controlled trial assessing the effects of 20 Hz rTMS in patients with AD, it was observed that brain activity exhibited a sustained modulation for a duration of up to 24 weeks following the initiation of the stimulation program (Koch et al., 2022). Therefore, rTMS poses as a potential technique to modulate memory and associated neurophysiological over an extended period. Nevertheless, it is necessary to conduct further investigations to comprehensively evaluate the long-term effectiveness of the rTMS and identify the optimal population to focus on.

### tDCS

Our tDCS analysis included 10 studies and revealed a significant improvement in memory abilities in AD patients. The subgroup analysis suggested that tDCS showed greater effectiveness when applied to temporal regions as opposed to frontal regions. In our analysis of tDCS studies, two of the selected studies employed a single-session crossover design to compare the effects of temporal stimulation with frontal stimulation. Liu et al. (2020) reported significant improvement on a 2-back task following temporal tDCS, as compared to frontal and sham stimulations. In another study, Boggio et al. (2009) observed an enhancement of recognition memory following the application of frontal and temporal tDCS in comparison with a sham condition.

The potential benefit of focusing on temporal areas rather than frontal areas in AD could be explained by the alterations in the functional connectivity observed in the medial temporal lobe during the early stages of the AD continuum (Berron et al., 2020). Moreover, these changes also occur during memory processing in patients with AD, as indicated by reduced activations in the medial temporal lobe and superior temporal/inferior parietal associative areas in comparison to healthy controls (Rémy et al., 2005). These findings suggest a potential reason for the greater efficacy of temporal tDCS in individuals with AD compared to healthy controls. It appears that stimulating the DLPFC using tDCS is the most effective method for modulating cognitive functions, including memory, in healthy older adults (Indahlastari et al., 2021) and in patients with Parkinson's disease (Suarez-García et al., 2020). Therefore, it appears that patients with AD experience more advantages from temporal tDCS, whereas healthy individuals or patients with other neurodegenerative disorders may find tDCS in different brain regions more advantageous. In addition, the lack of effect of frontal tDCS compared to the improvement observed following frontal rTMS could possibly be attributed to distinct neurophysiological effect of both techniques on functional networks. For instance, in one study with older adults, high-frequency frontal rTMS increased functional connectivity within DMN, which was accompanied with an improvement in memory capabilities (Cui et al., 2022). On the other hand, research on tDCS studies has not yielded conclusive evidence on its effects on the DMN, and it has primarily been examined in younger adults (Coulborn & Fernández-Espejo, 2022; Peña-Gómez et al., 2012). Nevertheless, it is important to acknowledge that although DMN is a functional network altered in AD, it is not the only network that is impacted (Agosta et al., 2012), and as such future studies should probe the mechanistic effects of brain stimulation techniques in several brain networks.

Moreover, the tDCS effect size was not as large as the one observed in the rTMS analysis (SMD_tDCS_ = 0.20 vs. SMD_rTMS_ = 0.44). This discrepancy might be explained by differences between both techniques such as higher spatial resolution and neurophysiological specificity in rTMS (Priori et al., 2009). For instance, a study comparing the neurophysiological effects of both techniques showed that 10 Hz rTMS increased the cortical excitability in the stimulation region in comparison with tDCS (Simis et al., 2013). Besides that, we should also underscore that our risk of bias analysis revealed higher risk in the tDCS studies in comparison with rTMS, namely in randomization, allocation concealment, selective reporting, and rater blinding. However, the comparative effect of these two techniques on brain functioning in clinical populations is still not clear, necessitating additional investigation in future studies.

### Safety

In the current study, we also intended to analyze the safety of each technique in AD patients. A total of twelve studies analyzed the AEs in both techniques where the most prevalent AE was related to scalp sensations or discomfort. In general, every study reported minor and transient AEs that were tolerable in both techniques. Only one study using rTMS reported two dropouts (4 % of the study sample) due to persistent scalp discomfort and fatigue. Taken together, our findings suggest the safety of both techniques in the AD population in line with previous evaluations in other clinical populations (Bikson et al., 2016; Rossi et al., 2021). In specific, the transient AEs observed in our study are consistent with those reported in other studies in chronic pain Cardenas-Rojas et al. (2020), major depressive disorder (Perera et al., 2016), non-fluent aphasia (López-Romero et al., 2019) and in healthy subjects (Poreisz et al., 2007).

### Limitations

The current meta-analysis comprises a limited number of studies addressing the effect of tDCS and rTMS on memory abilities. Most studies evaluate the efficacy of both techniques in global cognition (Teselink et al., 2021; Xiu et al., 2023; Zhang et al., 2022), which makes it more difficult to comprehend how they affect a specific cognitive process. In particular, NIBS may have different effects depending on the stimulation parameters and underlying brain network because memory is a broad domain that may be divided into various subprocesses (e.g., STM, LTM, or WM).

Additionally, most of the studies considered in our meta-analysis enrolled AD participants based on the probable AD criteria from NINCDS–ADRDA (Dubois et al., 2007) (see Table D in Supplementary Materials). One significant limitation of this diagnostic approach is that it relies heavily on clinical outcomes, with minimal incorporation of biomarkers. However, the probable AD criteria from NINCDS–ADRDA demonstrated sensitivities and specificities larger than 80 % when identifying patients with AD compared to other neurodegenerative disorders (Dubois et al., 2007). Likewise, studies using only clinical criteria, such as the Diagnostic and Statistical Manual of Mental Disorders Fifth Edition (DSM-V), excluded anyone who had a diagnosis of a different neurodegenerative (Bystad et al., 2016; Jia et al., 2021; Wei et al., 2022). As a result, our sample of studies might comprise a heterogeneous population with distinct genetic and stochastic profiles and consequently divergent pathophysiology (Frisoni et al., 2022). Future research using NIBS on AD should prioritize identifying AD pathology to ensure accurate disease identification and a more uniform study population.

Lastly, only one of the tDCS studies examined the long-term effect, therefore, our meta-analysis did not include a memory follow-up evaluation in the tDCS analysis. Likewise, even though it was possible to test the follow-up evaluation in rTMS, the number of studies was also low and with distinct follow-up periods, which suggests future AD studies to address how both techniques might be able to enhance memory in the long-term.

### Conclusion

Overall, the current study showed that rTMS and tDCS can improve memory in AD patients. The rTMS yielded better results when administered in frontal regions, exhibiting a higher effect size compared to the tDCS, which showed greater efficacy when provided in temporal areas. However, the reason behind the superior efficacy of rTMS in frontal regions and tDCS in temporal regions is still not clear. Moreover, our results are consistent with prior meta-analyses testing the effect of both techniques on global cognition in AD. This is of particular interest because memory impairments are the most common symptom in AD, which can suggest that the similar effects observed in global cognition might rely mainly on memory ability and frontotemporal networks. Lastly, our analysis also suggests the safety of both techniques, as most of the studies examined reported minimal and transient side effects.

## Declaration of competing interest

The authors declare that they have no known competing financial interests or personal relationships that could have appeared to influence the work reported in this paper.

## Appendix Supplementary materials

Image, application 1
