# Supplementary material for: Efficacy and safety of repetitive Transcranial Magnetic Stimulation and transcranial Direct Current Stimulation in memory deficits in patients with Alzheimer's disease: Meta-analysis and systematic review
Source: Int J Clin Health Psychol. 2024 Mar 1;24(2):100452. doi: 10.1016/j.ijchp.2024.100452 (PMC10914562; doi:10.1016/j.ijchp.2024.100452)
Supplement: Supplementary file 1 [file mmc1.docx]

***Supplementary Materials***

**Table A.** Combination of keywords used in the search literature.

| **Databases** | **Date** | **Keywords** |
| --- | --- | --- |
| Pubmed | 7/10/2022 | ("Transcranial Direct Current Stimulation" OR "noninvasive brain stimulation" OR "non-invasive brain stimulation" OR "noninvasive brain stimulation" OR "transcranial direct current stimulation" OR “neuromodulation” OR “NIBS” OR “TDCS” OR "transcranial electrical stimulation" OR "transcranial stimulation" OR "Transcranial Magnetic Stimulation" OR "transcranial magnetic stimulation" OR “TMS” OR “Rtms”)  AND (“memory” OR “working memory” OR “WM” OR “recognition” OR “recall” OR “neuropsychological” OR “cognitive” OR “cognition”)  AND (“Alzheimer” OR “Alzheimer's” OR “Alzheimer's disease” OR “AD”) |
| Scopus | 7/10/2022 | ( TITLE-ABS-KEY ( {Transcranial Direct Current Stimulation} OR {noninvasive brain stimulation} OR {non-invasive brain stimulation} OR {noninvasive brain stimulation} OR {transcranial direct current stimulation} OR {neuromodulation} OR {NIBS} OR {TDCS} OR {transcranial electrical stimulation} OR {transcranial stimulation} OR {Transcranial Magnetic Stimulation} OR {transcranial magnetic stimulation} OR {TMS} OR {Rtms} ) AND TITLE-ABS-KEY ( {memory} OR {working memory} OR {WM} OR {recognition} OR {recall} OR {neuropsychological} OR {cognitive} OR {cognition} ) AND TITLE-ABS-KEY ( {Alzheimer} OR {Alzheimer's} OR {Alzheimer's disease} OR {AD} ) ) |
| Web of Science | 7/10/2022 | ALL=("Transcranial Direct Current Stimulation" OR "noninvasive brain stimulation" OR "non-invasive brain stimulation" OR "noninvasive brain stimulation" OR "transcranial direct current stimulation" OR “neuromodulation” OR “NIBS” OR “TDCS” OR "transcranial electrical stimulation" OR "transcranial stimulation" OR "Transcranial Magnetic Stimulation" OR "transcranial magnetic stimulation" OR “TMS” OR “Rtms”) AND ALL=(“memory” OR “working memory” OR “WM” OR “recognition” OR “recall” OR “neuropsychological” OR “cognitive” OR “cognition” ) AND ALL=(“Alzheimer” OR “Alzheimer's” OR “Alzheimer's disease” OR “AD”) |

**Table B.** Labels of the comparisons in each study, namely the different memory evaluations, or stimulation montage, or follow-up.

|  |  | a | b | c | d | e | f | g | h | i | j |
| --- | --- | --- | --- | --- | --- | --- | --- | --- | --- | --- | --- |
| tDCS | Bystad (2016) | CVLT-II Rec. | CVLT-II - DR | CVLT-II - IR |  |  |  |  |  |  |  |
|  | Liu (2020) | bifrontal | bitemporal |  |  |  |  |  |  |  |  |
|  | Boggio (2009) | frontal | temporal |  |  |  |  |  |  |  |  |
|  | Rasmussen (2021) | RBANS - DR | *RBANS - IR* |  |  |  |  |  |  |  |  |
|  | Cotelli (2014) | MT & ROCF | CT & ROCF | MT & RAVLT - DR | CT & RAVLT - DR | MT & RAVLT - IR | CT & RAVLT - IR | MT & RBMT - DR | CT & RBMT - DR | MT & RBMT - IR | CT & RBMT - IR |
|  | Im (2019) | *RCFT Rec.* | RCFT - DR | *RCFT - IR* | *SVLT rec.* | SVLT - DR | *SVLT - IR* |  |  |  |  |
| TMS | Jia (2021) | CDR | PVLT Dist.R | PVLT LDR | PVLT SDR | PVLT IR |  |  |  |  |  |
|  | Koch (2018) | RAVLT - DR | RAVLT - IR |  |  |  |  |  |  |  |  |
|  | Wu (2022) | AM | DS - backward | DS - forward | AVLT rec. | AVLT - DR | AVLT - IR | LMT - DR | LMT - IR |  |  |
|  | Bagattini (2020) | ROCF - DR | RAVLT - DR | RAVLT - IR | Story Recall |  |  |  |  |  |  |
|  | Wei(2022) | PVLT - DR | PVLT - SDR | PVLT - IR |  |  |  |  |  |  |  |
|  | Kumar (2020) | 14-days FU | 7-days FU |  |  |  |  |  |  |  |  |

***Legend:*** CVLT-II - California Verbal Learning Test-Second Edition; Rec. - Recognition; DR - delayed recall; IR - immediate recall; SDR- Short delay recall; LDR - Long delay recall; RBANS - Repeatable Battery for the Assessment of Neuropsychological Status; MT - motor training; CT - cognitive training; ROCF - Rey-Osterrieth complex figure; RAVLT - Rey Auditory Verbal Learning Test; RBMT - Rivermead Behavioural Memory Test; RCFT - Rey Complex Figure Test; CDR - Clinical Dementia Rating; Dist. - distractor; PVLT - Philadelphia Verbal Learning Test; AM - Associative memory; DS - Digit span; LMT - Logical Memory Test; FU - follow-up

**Table C.** List of references of the studies included in quantitative synthesis.

1. **tDCS studies**

Boggio, P. S., Khoury, L. P., Martins, D. C., Martins, O. E., De Macedo, E. C., & Fregni, F. (2009). Temporal cortex direct current stimulation enhances performance on a visual recognition memory task in Alzheimer disease. *Journal of Neurology, Neurosurgery & Psychiatry, 80*(4), 444-447.

Boggio, P. S., Ferrucci, R., Mameli, F., Martins, D., Martins, O., Vergari, M., ... & Priori, A. (2012). Prolonged visual memory enhancement after direct current stimulation in Alzheimer's disease. *Brain stimulation, 5*(3), 223-230.

Bystad, M., Grønli, O., Rasmussen, I. D., Gundersen, N., Nordvang, L., Wang-Iversen, H., & Aslaksen, P. M. (2016). Transcranial direct current stimulation as a memory enhancer in patients with Alzheimer’s disease: a randomized, placebo-controlled trial. *Alzheimer's research & therapy, 8(*1), 1-7.

Cespón, J., Rodella, C., Miniussi, C., & Pellicciari, M. C. (2019). Behavioural and electrophysiological modulations induced by transcranial direct current stimulation in healthy elderly and Alzheimer’s disease patients: a pilot study. *Clinical Neurophysiology, 130*(11), 2038-2052.

Cotelli, M., Manenti, R., Brambilla, M., Petesi, M., Rosini, S., Ferrari, C., ... & Miniussi, C. (2014). Anodal tDCS during face-name associations memory training in Alzheimer's patients. *Frontiers in aging neuroscience, 6*, 38.

Ferrucci, R., Mameli, F., Guidi, I., Mrakic-Sposta, S., Vergari, M., Marceglia, S. E. E. A., ... & Priori, A. (2008). Transcranial direct current stimulation improves recognition memory in Alzheimer disease. *Neurology, 71*(7), 493-498

Gangemi, A., & Fabio, R. A. (2020). Transcranial direct current stimulation for Alzheimer disease. *Asian Journal of Gerontology and Geriatrics, 15*(1), 5-9.

Im, J. J., Jeong, H., Bikson, M., Woods, A. J., Unal, G., Oh, J. K., ... & Chung, Y. A. (2019). Effects of 6-month at-home transcranial direct current stimulation on cognition and cerebral glucose metabolism in Alzheimer's disease. *Brain stimulation, 12*(5), 1222-1228.

Liu, C. S., Herrmann, N., Gallagher, D., Rajji, T. K., Kiss, A., Vieira, D., & Lanctôt, K. L. (2020). A pilot study comparing effects of bifrontal versus bitemporal transcranial direct current stimulation in mild cognitive impairment and mild Alzheimer disease. *The Journal of ECT, 36(*3), 211.

Rasmussen, I. D., Boayue, N. M., Mittner, M., Bystad, M., Grønli, O. K., Vangberg, T. R., ... & Aslaksen, P. M. (2021). High-definition transcranial direct current stimulation improves delayed memory in Alzheimer’s disease patients: a pilot study using computational modeling to optimize electrode position. *Journal of Alzheimer's Disease, 83*(2), 753-769.

1. **TMS studies**

Bagattini, C., Zanni, M., Barocco, F., Caffarra, P., Brignani, D., Miniussi, C., & Defanti, C. A. (2020). Enhancing cognitive training effects in Alzheimer’s disease: rTMS as an add-on treatment. *Brain Stimulation, 13*(6), 1655-1664.

Jia, Y., Xu, L., Yang, K., Zhang, Y., Lv, X., Zhu, Z., ... & Chen, W. (2021). Precision repetitive transcranial magnetic stimulation over the left parietal cortex improves memory in Alzheimer’s disease: A randomized, double-blind, sham-controlled study. *Frontiers in Aging Neuroscience, 13*, 693611.

Kumar, S., Zomorrodi, R., Ghazala, Z., Goodman, M. S., Blumberger, D. M., Daskalakis, Z. J., ... & Rajji, T. K. (2020). Effects of repetitive paired associative stimulation on brain plasticity and working memory in Alzheimer’s disease: a pilot randomized double-blind-controlled trial. *International Psychogeriatrics*, 1-13.

Koch, G., Bonnì, S., Pellicciari, M. C., Casula, E. P., Mancini, M., Esposito, R., ... & Bozzali, M. (2018). Transcranial magnetic stimulation of the precuneus enhances memory and neural activity in prodromal Alzheimer's disease. *Neuroimage, 169*, 302-311.

Turriziani, P., Smirni, D., Mangano, G. R., Zappalà, G., Giustiniani, A., Cipolotti, L., & Oliveri, M. (2019). Low-frequency repetitive transcranial magnetic stimulation of the right dorsolateral prefrontal cortex enhances recognition memory in Alzheimer’s disease. *Journal of Alzheimer's Disease, 72*(2), 613-622.

Rutherford, G., Lithgow, B., & Moussavi, Z. (2015). Short and long-term effects of rTMS treatment on Alzheimer's disease at different stages: a pilot study. *Journal of experimental neuroscience, 9*, JEN-S24004.

Wei, L., Zhang, Y., Wang, J., Xu, L., Yang, K., Lv, X., ... & Chen, W. (2022). Parietal-hippocampal rTMS improves cognitive function in Alzheimer's disease and increases dynamic functional connectivity of default mode network. *Psychiatry Research*, 315, 114721.

Wu, X., Ji, G. J., Geng, Z., Wang, L., Yan, Y., Wu, Y., ... & Wang, K. (2022). Accelerated intermittent theta-burst stimulation broadly ameliorates symptoms and cognition in Alzheimer's disease: A randomized controlled trial. Brain Stimulation, 15(1), 35-45.

Yao, Q., Tang, F., Wang, Y., Yan, Y., Dong, L., Wang, T., ... & Shi, J. (2022). Effect of cerebellum stimulation on cognitive recovery in patients with Alzheimer disease: A randomized clinical trial. *Brain Stimulation*, 15(4), 910-920.

**Table D.** Characteristics of the TMS studies regarding stimulation, methodology, and sample.

| **First author (Year)** | **rTMS group** | **Control group** | **Study Design** | **rTMS intensity (%)** | **rTMS frequeny (Hz)** | **Number of pulses** | **Coil position** | **Number of Sessions** | **Cognitive Training** | **Medication** | **Follow-up** | **Safety Evaluation** | **Diagnostic criteria** | **Cognitive Inclusion criteria** |
| --- | --- | --- | --- | --- | --- | --- | --- | --- | --- | --- | --- | --- | --- | --- |
| Kumar (2020) | 16 | 16 | Parallel | NR | 0.1 | 180 | left DLPFC | 20 | No | Both | 14-days | Yes | NINCDS-ADRDA | MMSE > 17 |
| Rutherford (2015) | 6 | 6 | Crossover | 90-100 | 20 | 2000 | left and right DLPFC | 13 | Yes | Yes | No | No | Neuropsychiatrist or neurologist | 5 > MOCA > 26 |
| Bagattini (2020) | 27 | 23 | Parallel | 100 | 20 | 2000 | left DLPFC (F3) | 20 | Yes | Both | 2-months | Yes | NINCDS-ADRDA | MMSE > 16 & 0.5 < CDR < 2 |
| Yao (2022) | 15 | 12 | Parallel | 90 | 5 | 2000 | bilateral cerebellum | 20 | No | Both | 2-months | Yes | NIA-AA 2018 | MMSE > 16 |
| Koch (2018) | 14 | 14 | Crossover | 100 | 20 | 1600 | Precuneus | 10 | No | NR | No | No | prodromal AD (Dubois et al., 2016) | - |
| Jia (2021) | 35 | 34 | Parallel | 100-110 | 10 | 800 | MNI coordinates x = −47, y = −68, z = + 36 (left parietal site) | 10 | No | Yes | No | Yes | DSM-V | 0.5 < CDR <2 |
| Wei (2022)* | 21 | 20 | Parallel | 100-110 | 10 | 800 | MNI coordinates x = −47, y = −68, z = + 36 (left parietal site) | 11 | No | Yes | 12-weeks* | Yes | DSM-V | 0.5 < CDR <2 |
| Wu (2022) | 24 | 23 | Parallel | 70 | 5 | 1800 | left DLPFC (MNI coordinates 38 44 26) | 14 | No | Yes | 10-weeks | Yes | NINCDS-ADRDA | 10 < MMSE < 27 & CDR < 2 |
| Turriziani (2019) | 7 | 7 | Parallel | 90 | 1 | 60 | F3 & F4 | 10 | Yes | Yes | 1-month | No | NINCDS-ADRDA | - |

**Table E.** Characteristics of the tDCS studies regarding stimulation, methodology, and sample.

* This study had three experimental conditions: anodal tDCS with cognitive training, anodal tDCS with motor training, and sham with cognitive training.

| **First author (Year)** | **tDCS group** | **Control group** | **Study Design** | **tDCS intensity (mA)** | **tDCS density (mA/cm^2^)** | **Duration (min)** | **Active Electrode** | **Return Electrode** | **Electrode Size (cm^2^)** | **Number of Sessions** | **Cognitive Training** | **Medication** | **Follow-up** | **Safety evaluation** | **Diagnostic criteria** | **Cognitive Inclusion Criteria** |
| --- | --- | --- | --- | --- | --- | --- | --- | --- | --- | --- | --- | --- | --- | --- | --- | --- |
| Ferrucci (2008) | 10 | 10 | Crossover | 1.5 | 0.06 | 30 | P3-T5 and P6-T4 | right deltoid muscle | 25 | 1 | No | Yes | No | No | NINCDS-ADRDA | MMSE > 20 |
| Gangemi (2020) | 26 | 15 | Parallel | NR | NR | NR | F3 and F7 | right supraorbital area | 35 | 1 | No | Yes | No | Yes | NINCDS-ADRDA | 12 < MMSE < 25 |
| Boggio (2009) | 10 | 10 | Crossover | 2 | 0.06 | 30 | F3 or T7 | right supraorbital area | 35 | 1 | No | Both | No | Yes | NINCDS/ADRDA | 12 < MMSE < 25 |
| Im (2019) | 18 | 11 | Parallel | 2 | 0.07 | 30 | F3 | F4 | 28.27 | multiple (NR) | NR | Yes | No | No | NINCDS-ADRDA | 0.5 < CDR < 1 |
| Cotelli (2014) | 12+12 | 12 | Parallel | 2 | 0.08 | 25 | left DLPFC | right deltoid muscle | 25 (return 60) | 5 | Yes* | Yes | 3 & 6-months | No | NINCDS-ADRDA | - |
| Cespón (2019) | 12 | 12 | Crossover | 1.5 | 0.09 | 13 | F3 | right shoulder | 16 (50) | 1 | Yes | Yes | No | No | NINCDS-ADRDA | MMSE > 16 |
| Liu (2020) | 17 | 17 | Crossover | 2 | 0.06 | 20 | F3 & F4 or T3 & T4 | Iz | 35 | 1 | No | No | No | Yes | DSM-V | MOCA > 12 |
| Boggio (2012) | 15 | 15 | Crossover | 2 | 0.06 | 30 | T3 and T4 | right deltoid muscle | 35 | 5 | No | No | No | Yes | NINCDS-ADRDA and DSM-V | MMSE > 15 |
| Rasmussen (2021) | 10 | 9 | Parallel | 2 |  | 20 | F3 | anode surrounded by four cathodes | 1.13 | 6 | No | NR | No | Yes | NINCDS-ADRDA | MMSE > 17 |
| Bystad (2016) | 12 | 13 | Parallel | 2 | 0.06 | 30 | T3 | Fp2 | 35 | 6 | No | NR | No | Yes | NINCDS-ADRDA | MMSE > 18 |

**Figure A.** Funnel plots in each pooled effect analysis, namely, tDCS, TMS, and TMS follow-ups.

TMS follow-ups studies

TMS studies

tDCS studies


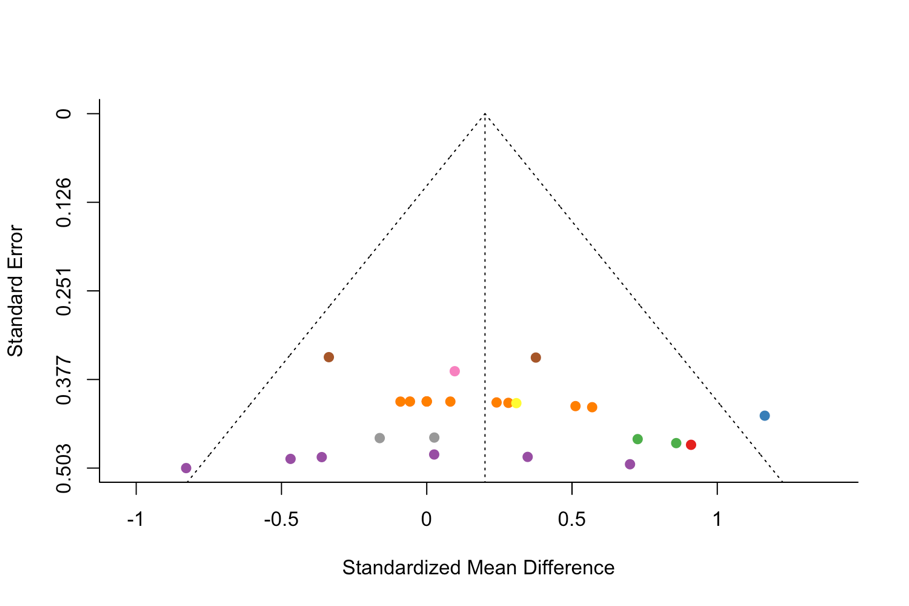

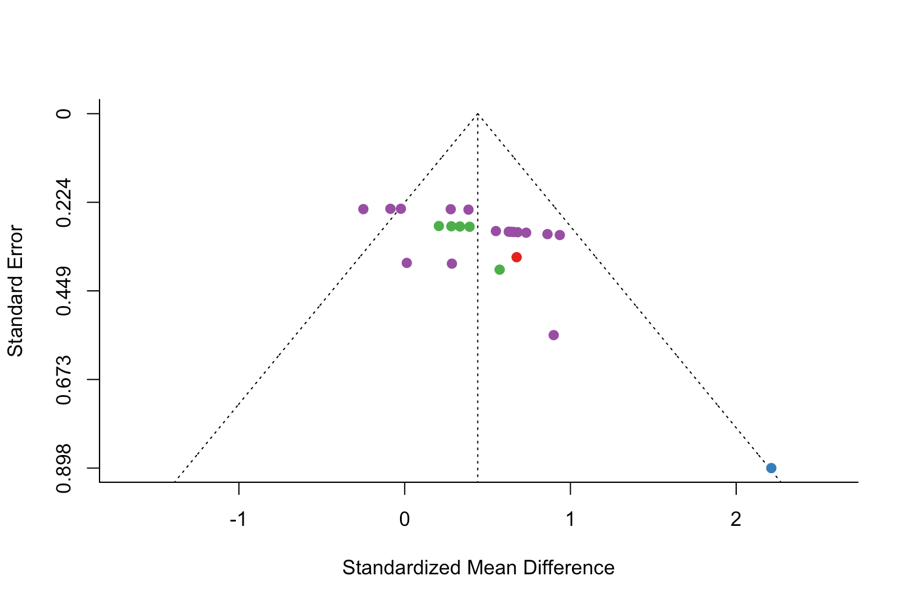

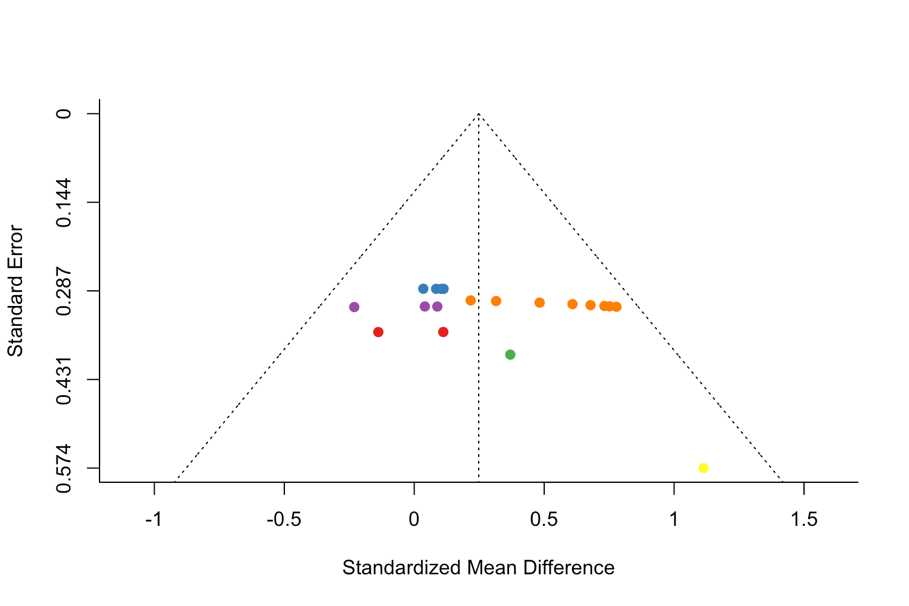


**Figure B.** Risk of bias assessment for each tDCS study and domain using the Cochrane tool.

**
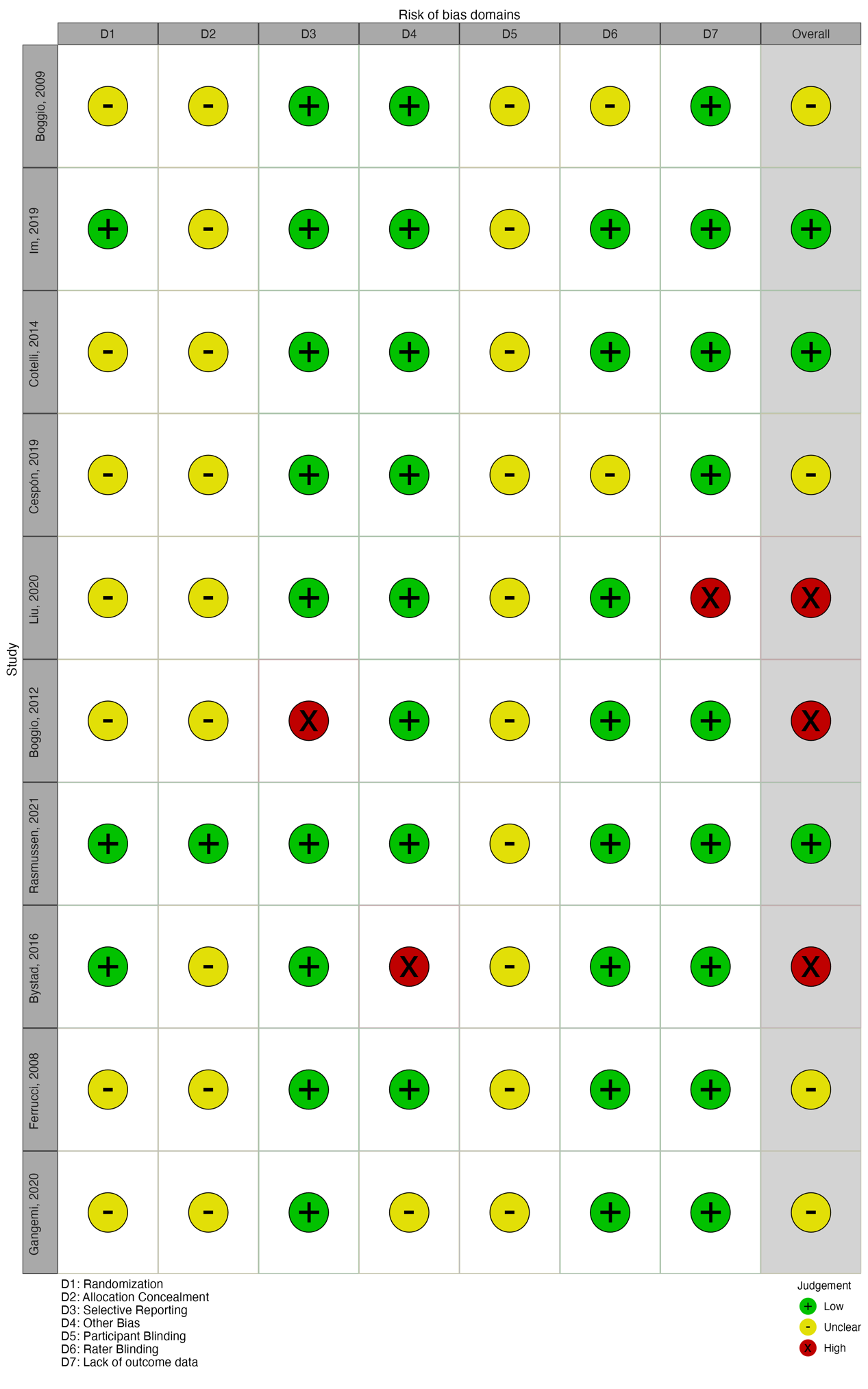
**

**Figure C.** Overall results of the risk of bias assessment for all tDCS studies. **
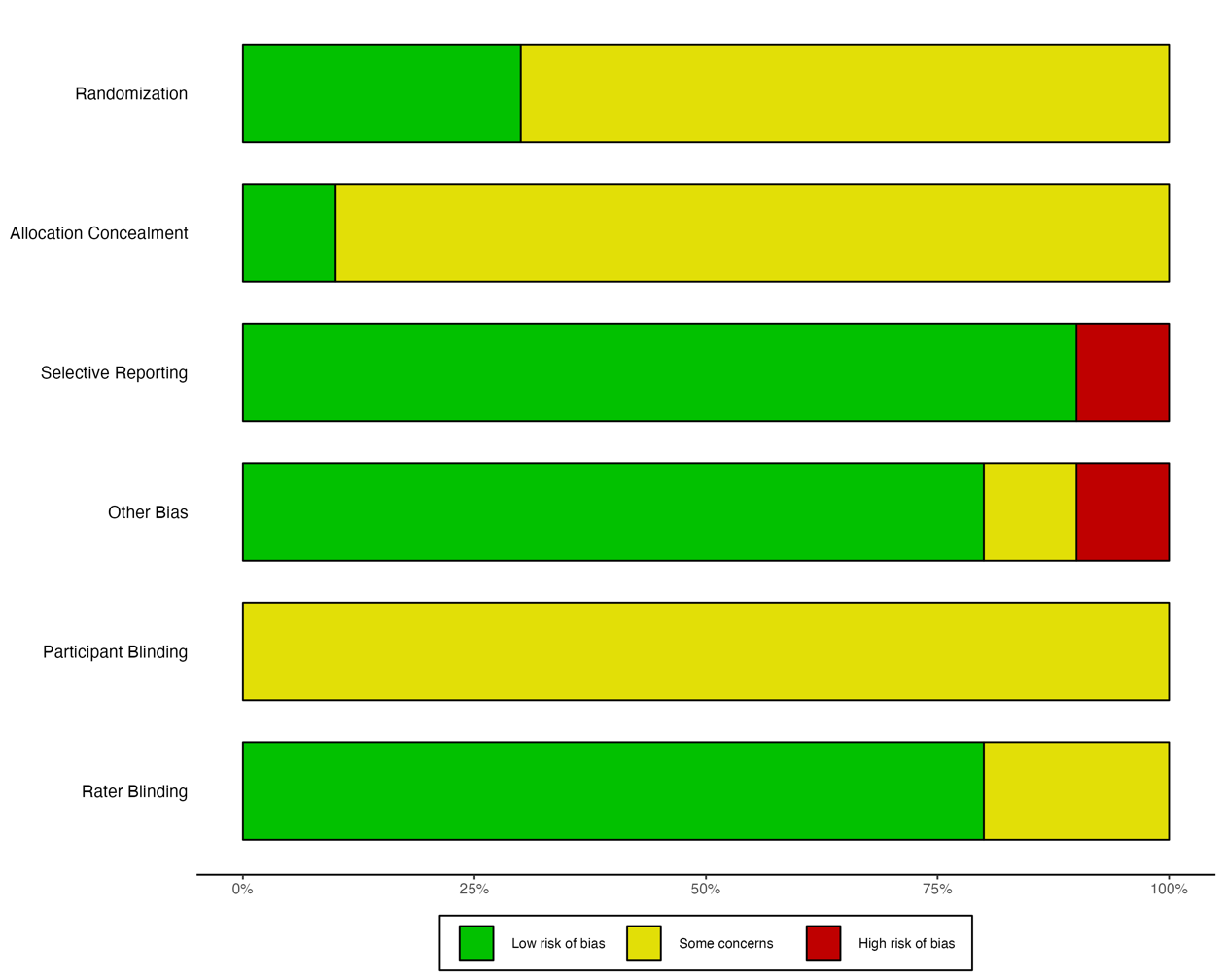
**

**Figure D.** Risk of bias assessment for each TMS study and domain using the Cochrane tool.


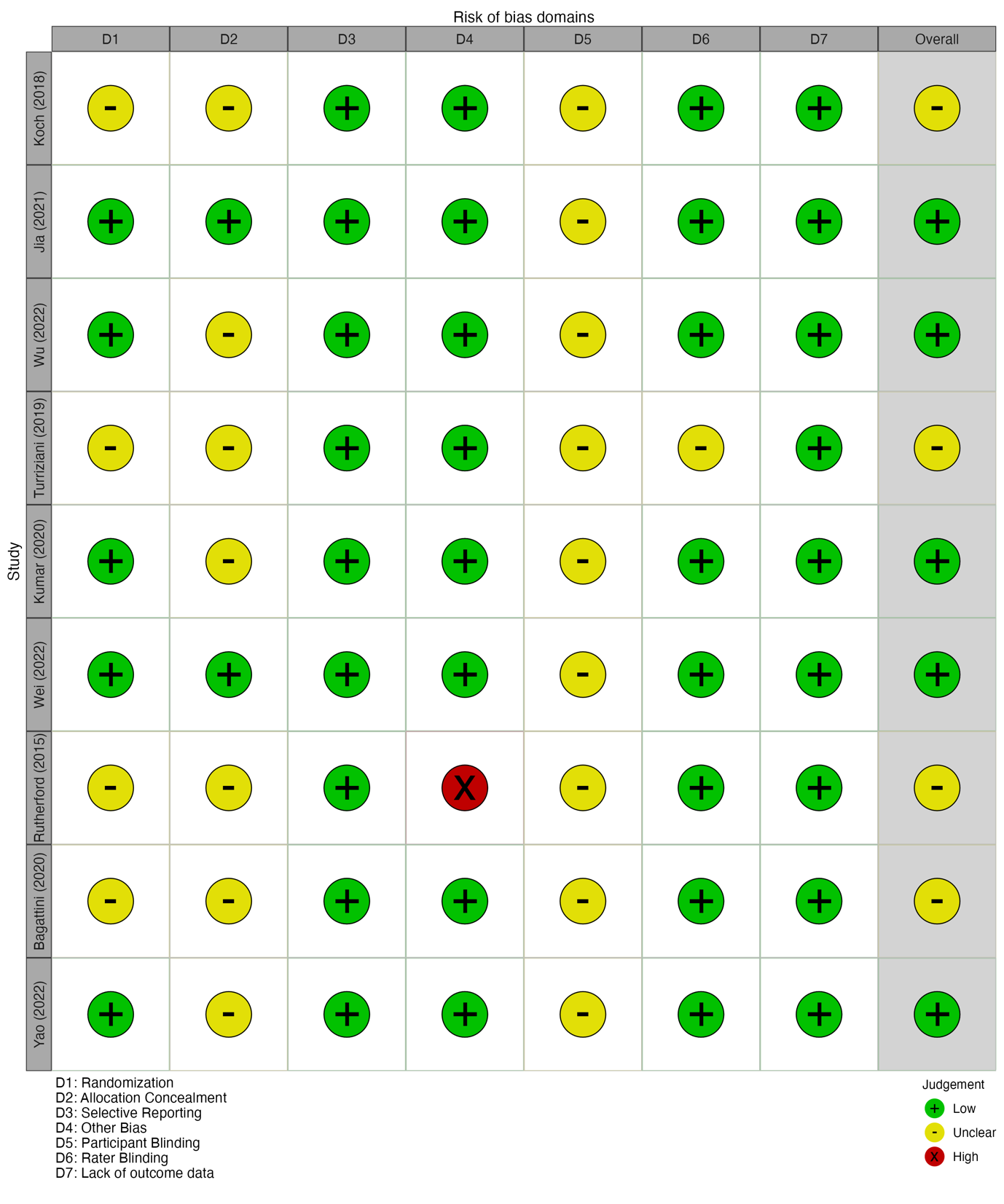


**Figure E.** Overall results of the risk of bias assessment for all TMS studies.

**
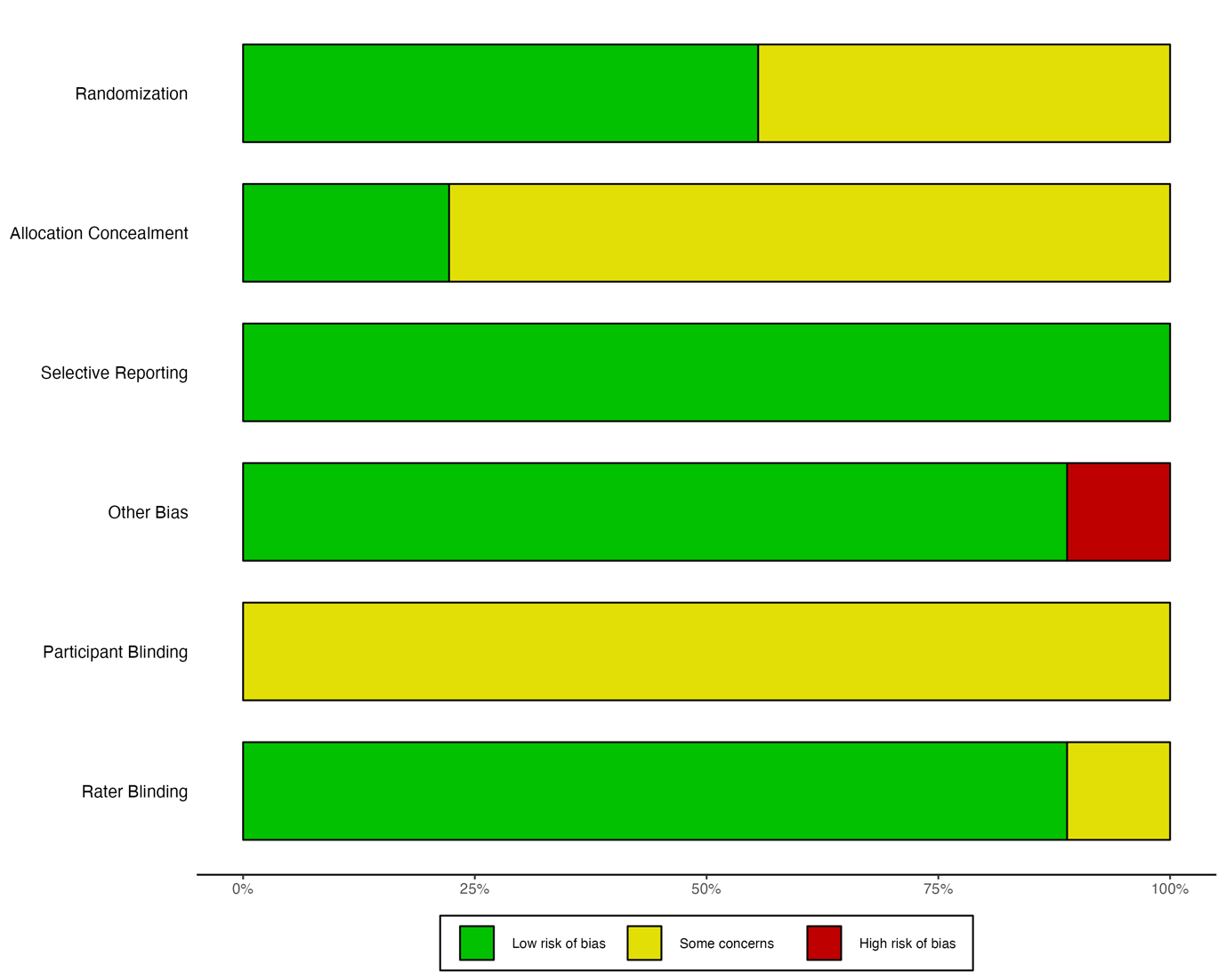
**
